# A Wireless Gamma-Ray Monitoring System for Cemented Radwaste Drums

**DOI:** 10.3390/s24072332

**Published:** 2024-04-06

**Authors:** Mauro Romoli, Michele Di Giovanni, Paolo Di Meo, Antonio Pandalone, Claudio Principe, Carlo Sabbarese, Antonio D’Onofrio, Karel Prchal, Jakub Záruba, Paolo Finocchiaro

**Affiliations:** 1 INFN Sezione di Napoli, 80126 Napoli, Italy; romoli@na.infn.it (M.R.); michele.digiovanni@unicampania.it (M.D.G.); pdimeo@na.infn.it (P.D.M.); pandalone@na.infn.it (A.P.); claudio.principe@na.infn.it (C.P.); carlo.sabbarese@unicampania.it (C.S.); antonio.donofrio@unicampania.it (A.D.); 2 Department of Mathematics and Physics, University of Campania “Luigi Vanvitelli”, 81100 Caserta, Italy; 3 UJV Řež, Husinec, 250 68 Prague, Czech Republic; karel.prchal@ujv.cz (K.P.); jakub.zaruba@ujv.cz (J.Z.); 4 INFN Laboratori Nazionali del Sud, 95125 Catania, Italy

**Keywords:** wireless radiation sensor, gamma-ray counter, radwaste monitoring, cemented radwaste drum

## Abstract

In the framework of the PREDIS EU project, a wireless battery-operated gamma-ray detection system was developed in order to provide a medium-to-long-term monitoring system for radioactive waste drums. It was initially proposed to monitor the gamma radioactivity outcoming from steel drums containing cemented radwaste, even though it could be usefully employed in a wider range of applications. Gamma rays are penetrating and convey information from the drum's internal structure, as the count rate measured on the surface depends on the thickness and density of the crossed materials. A number of sensors arranged around a drum, typically four units, provide indications of the emission anisotropy, and any sensitive change in the measured count rate would hint at some anomaly, thus triggering a suitable inspection by operators.

## 1. Introduction

The PREDIS (predisposal management of radioactive waste) Euratom project is aimed at the development and implementation of activities for the predisposal treatment of radioactive waste streams other than nuclear fuel and high-level radioactive waste [1]. Within Work Package 7, the project contemplates testing and evaluating innovative tools and techniques in cemented waste handling and predisposal storage and, in particular, to demonstrate the feasibility of medium-to-long-term monitoring by means of low-cost radiation sensors to be installed around the radwaste drums as an alternative to more conventional procedures by operators [2]. A periodic automatic check of the radiation levels around radwaste drums could represent an added value with respect to safety and security, with recorded streams of count rate data providing a useful tool for the early detection of possible anomalies or tampering with the drums. The enhancement in terms of transparency is noteworthy as well [3,4,5].

Neutron [6] and gamma [7] radiation sensors for radwaste drum monitoring have already been developed and have proven feasible within the EU MICADO project [8]. However, the use of a wired online monitoring configuration for a real radwaste storage site would be a limitation, and this is why a wireless solution should be aimed at addressing the issue in [9]. In this paper, we describe the development of a wireless sensor, featuring a gamma-ray counter and a compact front-end and data acquisition electronics box, to be easily installed on a drum. The same gamma sensor of ref. [7] was employed, based on a scintillating fiber [10] whose output light is collected by a Silicon PhotoMultiplier (SiPM) [11,12,13,14,15] photosensor at each end. The two SiPMs are connected to two newly developed amplifiers, followed by analog-to-digital converters controlled by an Expressif ESP32 microcontroller [16]. Each box, operated on batteries, is connected wirelessly to a private sub-network exposed by a WiFi router and can be set to sleep and woken up for measurement periodically or on demand by means of a Low Energy Bluetooth link. A PC/Server with facilities for electronics and data management is also connected to the same sub-network.

Following a set of bench tests with low-activity laboratory sources, a demonstration was set up at UJV-REZ to prove the feasibility and validity of our solution. A suitable cemented mockup was built to insert a high-activity gamma source and test the sensors in a realistic environment. In Section 2, we describe the detector, front-end electronics, data acquisition, transmission, laboratory tests, and software features, along with the cemented mockup structure and its related simulation. In Section 3, we describe the system demonstration, i.e., the installation setup and tuning, and finally, the measurement results.

## 2. Materials and Methods

### 2.1. The Detector

**Figure 1:**
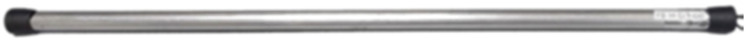
The SciFi detector, consisting of a 3-mm-diameter and 80-cm long scintillating fiber coupled at each end to a SiPM, enclosed in an aluminum pipe.

**Figure 2:**
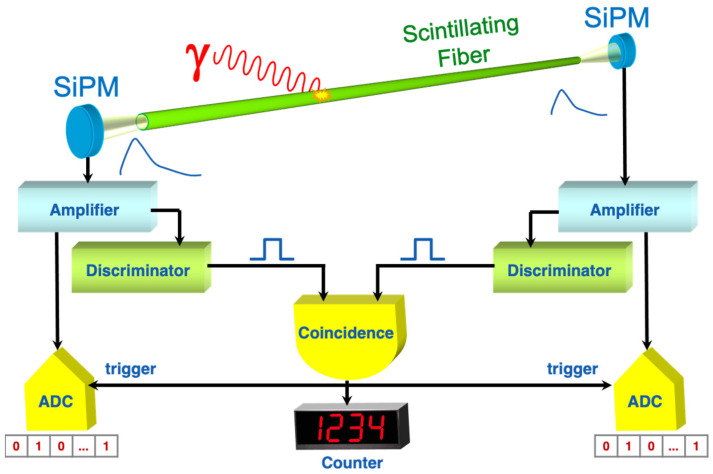
Sketch of the logical operating principle of a SciFi gamma-ray detector.

**Figure 3:**
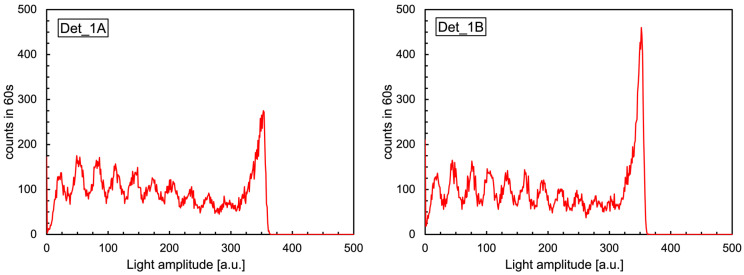
Typical amplitude spectra of the signals from the SiPMs at the two ends of a SciFi sensor. See the text for details.

Each detection unit consists of a Scintillating Fiber (SciFi) gamma-ray detector [7] coupled to its front-end electronics and data acquisition and transmission, which are allocated inside a dedicated box. The SciFi features a 3-mm-diameter and 80-cm long scintillating optical fiber [10] coupled at each end to a MicroFC-30035-SMT SiPM produced by ON Semiconductor [11]. A SciFi unit is shown in Figure 1, with the logical operating principle sketched in Figure 2. The typical amplitude spectra of the signals from the SiPMs at the two ends of a SciFi sensor are shown in Figure 3. The scintillation light produced by the fiber, when exposed to a gamma-ray source, gives rise to the multi-peak structure reflecting the discrete numbers of detected photons [12,13,14,15]. A suitable threshold set on the discriminators, along with the required coincidence between the signals at the two detector ends, suppresses the spurious noise [7]. The large peak around channel 350 is due to the saturation of the amplifier and does not represent a problem as the SciFi is just a radiation counter.

### 2.2. Front-End Electronics

The main guidelines for the development of the readout electronics board were as follows:

- Modularity, allowing for easy maintenance and the possibility to use the same electronics, with minor modifications, coupled to different detectors and in different scenarios.
- Low power consumption, obtained by choosing proper components and keeping the system in sleeping mode as long as possible, except during the measurement and data transmission phases.
- Low cost, by employing common components present on the market and reducing, when possible, the Research & Development costs.

**Figure 4:**
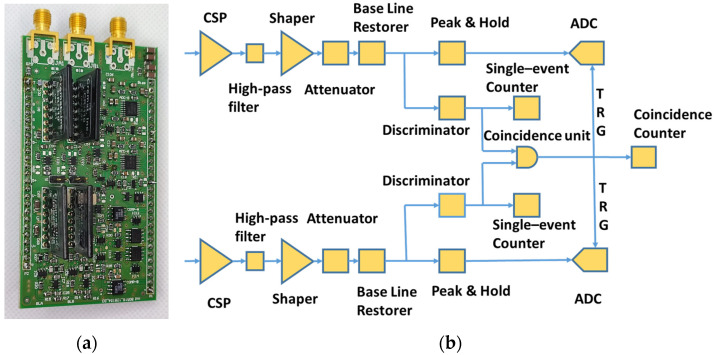
(**a**) The detector interface board (front-end board). (**b**) Block scheme of the board.

The treatment of the analog signals coming from the detector has been demanded to a separate mezzanine board (Figure 4a) handling two input channels. Each channel contains the following in sequence:

- Charge-sensitive pre-amplifier CREMAT CR-110 rev.2.01.
- High-pass filter.
- Shaper CREMAT CR-200-1µs.
- Attenuator with a factor of 0.05.
- Base-line restorer circuit (τ ≈ 700 µs).

Following that, each line is split in two. On one side, there is a threshold discriminator, with a threshold ranging from 0 mV to 4096 mV, with a 1 mV resolution; on the other side, there is a resettable Peak and Hold circuit, followed by a 14-bit programmable SAR ADC that is high-speed and single-supply (Texas Instruments ADS8675). A block scheme of the front-end board is shown in Figure 4b.

The signals on the two channels are analyzed individually and, when they are above the selected threshold, counted separately. Moreover, if they occur in coincidence within a time window of 2 μs, these events are counted as well, and their amplitudes are converted to digital and acquired. The single channel counters provide indications about the operation of the SiPMs and their noise level, while the coincidence counter provides the number of physical radiation detection events.

The bias voltage for the two SiPMs is common and supplied by an EMCO DC-DC converter (AG02P-5) capable of providing a floating voltage ranging from 0 to 200 V. In our case, we set the full scale to 50 V and used a 12-bit DAC to generate the desired voltage. In order to reduce the power consumption, the bias voltage is set and measured after wake-up directly at the DC-DC converter output by means of a 12-bit ADC, with a resolution of the order of 0.1% or less. Some filters were added to clean the output bias.

Both the mezzanine front-end board and the bias module are mounted on sockets on the main board, ensuring a quick and easy replacement in case of failure or to be employed with other kinds of detectors.

### 2.3. Data Acquisition and Transmission

The main board contains a microcontroller ESP32 (Espressif ESP32-WROVER-E), featuring a 3.3 V power supply, 240 MHz Dual Core CPU, 520 kB SRAM, 8 MB flash memory, and 8 MB PSRAM (both Quad SPI), devoted to the management of all the features related to the data collection and transmission. We opted for a system having built-in WiFi capability (802.11 b/g/n up to 150 Mbps), adding to the main board a separate module for the cabled Ethernet connection (WIZnet WIZ820io). The system has been designed to be constantly in standby mode in order to minimize the power consumption. The system wake-up for the scheduled measurements is obtained by means of a Real-Time Clock (RTC Maxim Integrated DS3231) mounted on a socket on the main board and is completely programmable. In the present configuration, following each measurement, the system establishes a WiFi contact with the PC/Server, uploads the data files, downloads the possibly modified scheduling for the next measurements, and returns to standby mode.

This automatic procedure can be overcome, whenever necessary, by sending a Bluetooth wake-up signal to the ESP32. In such a case, a forced system wake-up occurs, which starts an immediate measurement and connects the system to the PC/Server for further instructions. To make this possible, a Low Energy Bluetooth module (Microchip Technology Inc., RN4870/71-BLE) was installed on the main board. This component always remains active, with a negligible power consumption of the order of a few μW. In case of different future needs, the system modularity will allow the replacement of this BLE, currently limited to a maximum distance of 10–20 m, with different wake-up circuits having higher performances.

**Figure 5:**
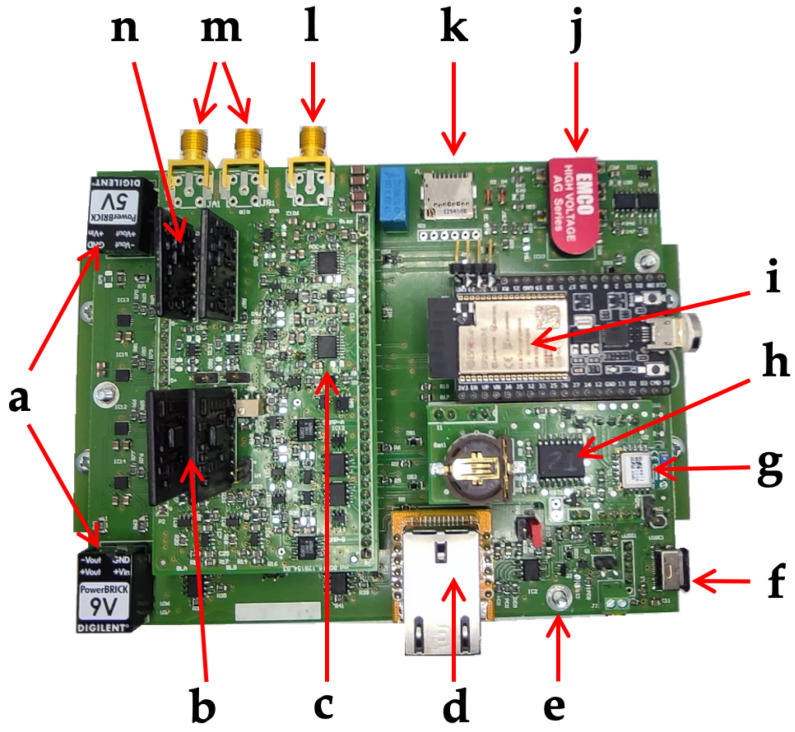
The main board, with an indication of the main components, including the piggy-back front-end board. (a) Power supply (9 V and 5 V). (b) Shapers. (c) ADCs. (d) Ethernet controller. (e) 3-position switch: power on with battery, power off, power on with USB. (f) USB bias. (g) Low Energy Bluetooth. (h) RTC. (i) Microcontroller. (j) HV generator. (k) Micro-SD card. (l) Voltage bias to SciFi. (m) SciFi signal inputs. (n) Charge-sensitive pre-amplifiers.

**Figure 6:**
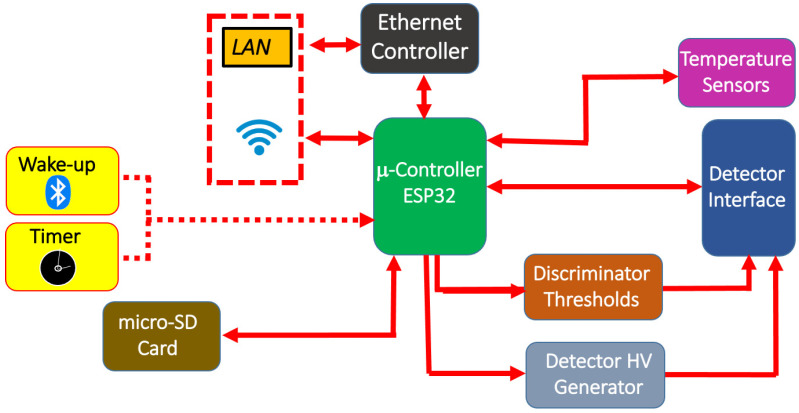
Block scheme of the main board with the interactions between the various devices and the microcontroller.

The ESP32 also demanded the control of the DACs to set the discriminator thresholds and the detector bias, the read-out of two temperature sensors placed on the main board and on the mezzanine interface board, and the data storage on the micro-SD memory card. The SPI protocol was used for the communication between the ESP32 and the other devices, except for the RTC, which communicates via the I2C protocol. The ESP32 firmware was developed using the Wiring language. Figure 5 shows the main board with the indication of the main components; the piggy-back front-end board is visible as well. Figure 6 shows the block scheme of the different devices on the board connected to the ESP32 and managed by it.

The installation of this kind of configuration was found to be quite simple. Moreover, with minor software modifications, the employed ESP32 microcontroller could be easily adapted to different systems as stand-alone devices or as nodes of a more open configuration in an IoT (Internet-of-Things) environment or a Mesh-Network architecture.

The average current absorbed by the system during the measurement cycles and during the standby phase is 350 mA and 40 μA, respectively. As for the battery pack, allocated inside the electronics box below the main board, we employed eight type-C Ni-MH standard rechargeable batteries, each with a 5000 mAh nominal charge and 1.2 V output voltage. The eight batteries were connected as a series of two groups of four in parallel to provide a total nominal voltage of 4.8 V and a charge of 10,000 mAh.

**Figure 7:**
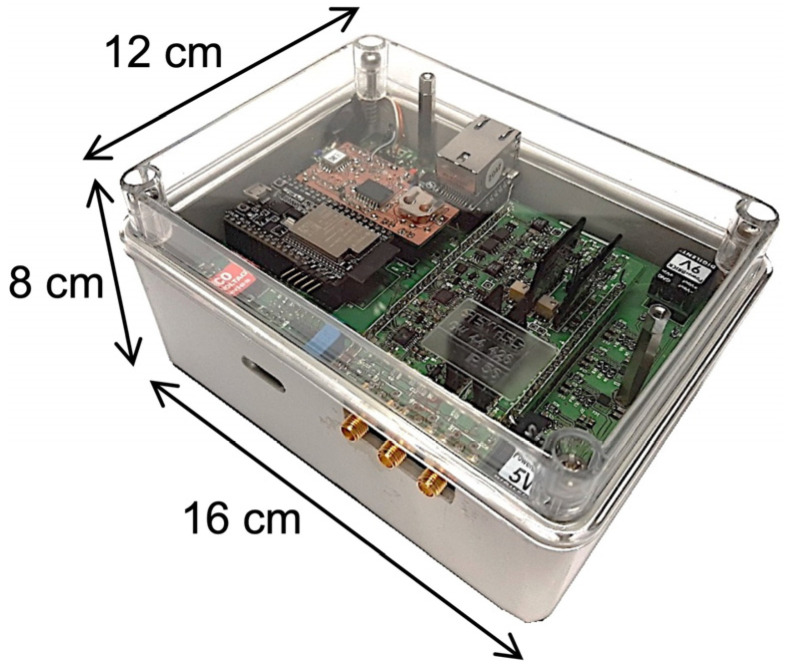
The electronics box containing front-end electronics, data acquisition and transmission hardware, ESP32 microcontroller, and batteries.

The weight of the electronics box is about 1.5 kg (essentially due to the battery pack), and a picture with the indication of the overall dimensions is reported in Figure 7. The three connectors on the side are for the SiPM common voltage bias and the two signal outputs. On the other side, there is a three-position switch: power off, power on with battery, and power on via USB port (not visible in Figure 7).

### 2.4. Test with a Laboratory Source

**Figure 8:**
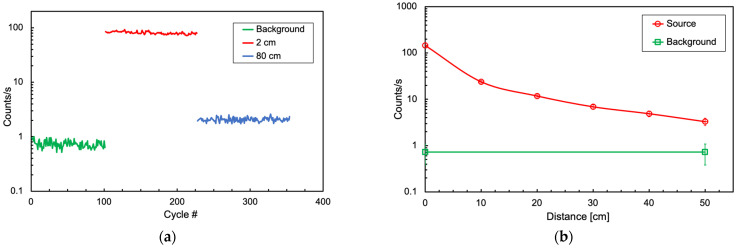
(**a**) Count rates measured in 365 runs of 60 s of data acquisition. Three different radioactive level conditions were tested: background, 2-cm, and 80-cm distance. (**b**) Count rate as a function of the source-detector distance. The background rate is indicated as a reference.

Several laboratory tests were performed using a calibration ^60^Co gamma source (157 kBq activity) to assess the performances and the stability of the electronics coupled to a SciFi detector. The source was a 1-mm-diameter grain encapsulated in a 20 × 10 × 2 mm^3^ transparent plastic tablet. Figure 8a shows the count rates measured in 365 runs, corresponding to one year, with one 60-s acquisition cycle per day. Three different radioactive level conditions were tested: background, near source (2 cm), and far source (80 cm). In Figure 8b, we show the count rate as a function of the source-detector distance, with the background rate shown as a reference.

**Figure 9:**
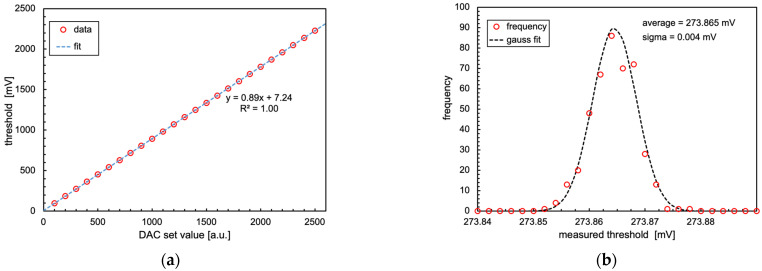
(**a**) Measured discriminator threshold versus value set on the DAC. Each point is the average of 400 measurements. The very small error bars are inside the symbols. (**b**) Example histogram of 400 threshold measurements and a Gaussian fit.

In Figure 9a we report the measured discriminator threshold (each value is the centroid of the distribution of 400 measurements) versus the value set by the DAC up to 2 V. The behavior is perfectly linear. Figure 9b shows an example of one such distribution, featuring a standard deviation of 4 µV.

**Figure 10:**
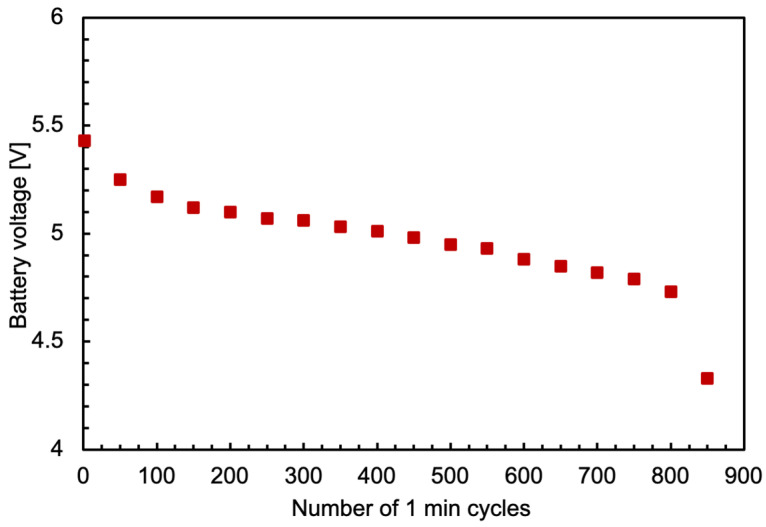
The battery pack output voltage as a function of the number of one-minute measurement cycles.

The battery pack discharge was evaluated throughout 850 measurement cycles by measuring its output voltage, and the results are plotted in Figure 10. The duration of each cycle was 60 s of acquisition plus 50 s for setup, stabilization, and data transmission. Considering a frequency of one cycle per day, an autonomy of approximately 2.5 years is ensured before having to recharge the battery pack.

### 2.5. Firmware and Software Development

The ESP32 microcontroller exposes an HTTP server through which remote instructions can be executed and parameters and wake-up alarms can be configured. The main features are briefly listed below.

- Dynamic device registration, allowing the registration of an unknown device.
- Programmatic parameter configuration, allowing the operator to define hardware and measurement parameters for each device interactively.
- Scheduling, where each device can measure at predefined times defined by one or more schedules.
- User Interface (UI), which allows an operator to interact with the devices and display all parameters.

**Figure 11:**
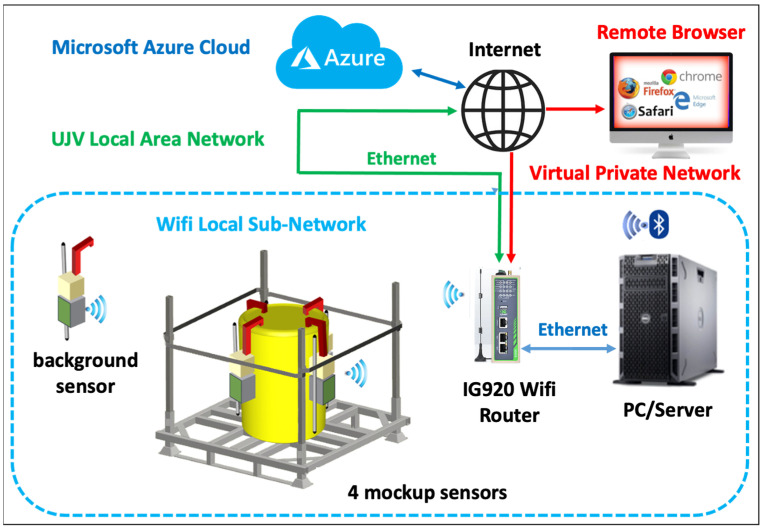
The interconnection scheme for the demonstration configuration installed at the UJV-REZ (Prague).

To expose those features in a safe and reliable manner, a containerized Flask/Python application server is deployed through Docker, exposing an HTTP application server and a front-end UI. These two services are accessible through a pre-configured Virtual Private Network (VPN). The interconnection scheme for the demonstration configuration installed at the UJV-REZ is shown in Figure 11. Four detection units were hung around a cemented mockup drum, with a fifth one in an adjacent room for background measurement. All the units, as well as the PC/Server, were connected to the local WiFi sub-network generated by a dedicated router with a public IP address on the UJV-REZ Local Area Network (LAN). The sub-network, and then the PC/Server and the five detection units were accessible both locally and remotely via a VPN. Indeed, this configuration enabled the authorized users to interact remotely with the server and the detection units to download the acquired data and allowed for possible setup changes when needed. This was effectively performed from Italy throughout the three-month test. 

### 2.6. The Mockup Drum

**Figure 12:**
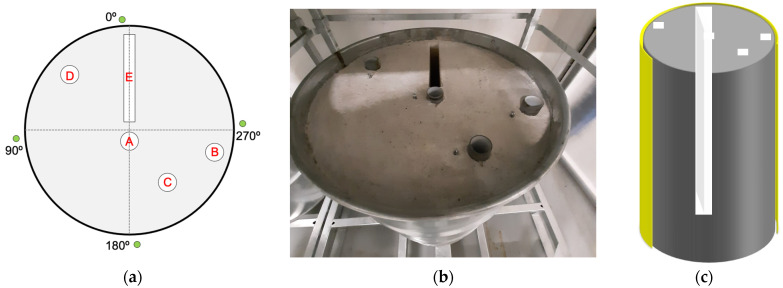
(**a**) Top view of the mockup scheme; the four green circles (at nominal 0°, 90°, 180°, and 270°) indicate the position of the SciFi sensors, and the white rectangle and circles represent the vertical holes in the concrete cylinder. (**b**) A top picture of the cemented mockup drum, with the lid removed. (**c**) A 3D sketch of the mockup inside; part of the steel skin is highlighted in yellow, the concrete is in grey, and the vertical holes are in light grey.

In the framework of the PREDIS WP7 project, a demonstration test was foreseen, aimed at proving the feasibility of a medium-to-long term monitoring of cemented radwaste drums. A 200 L concrete mockup, 820 mm high with 571 mm diameter and encased into a 1.2-mm thick galvanized steel drum, was built for the test. Its structure, shown in Figure 12, was initially meant to allow for the insertion of one or more radioactive sources in vertical holes running through the concrete cylinder’s full height, externally giving rise to a non-uniform radiation field to be detected and measured. The non-uniformity was foreseen to characterize the behavior of the sensors in response to such a field, showing that they would be capable of detecting asymmetries and/or deviations from the average, should anomalies occur to a real drum so equipped. Due to logistic reasons, a single configuration was chosen for the final demo, with a 165 MBq ^137^Cs gamma source placed into the quasi-central hole (A), and the drum was equipped with four SciFi sensors to monitor it during a one-to-three-month period. We remark that at the end of the test campaign, we discovered that the nominal SciFi positions were displaced from the effective ones due to a slightly rotated positioning of the drum lid during its hasty closure after inserting the high-activity source.

### 2.7. Mockup Simulation

**Figure 13:**
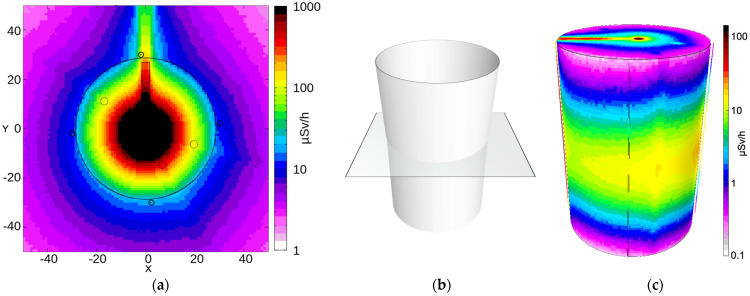
(**a**) Expected dose distribution on an XY cross-section at mid-height of the drum (40 cm < Z < 41 cm), according to the FLUKA simulation of the 165 MBq ^137^Cs gamma source placed at the same height inside the quasi-central hole of the mockup. All dimensions are in cm. (**b**) Indication of the cross-section plane. (**c**) Expected dose rate distribution on the drum surface in 3D. Three scintillating fibers are also visible.

**Figure 14:**
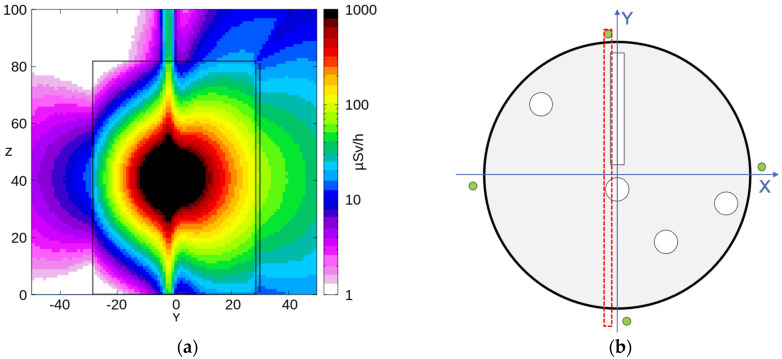
(**a**) Expected dose rate distribution on a YZ cross-section at −3 cm < X < −1 cm, which includes the 0° SciFi. (**b**) Indication of the cross-section plane.

**Figure 15:**
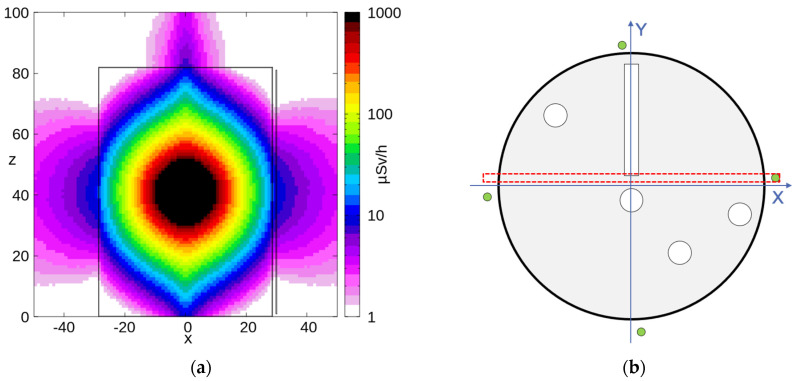
(**a**) Expected dose rate distribution on an XZ cross-section at 1 cm < Y < 3 cm. (**b**) 3D representation of the expected dose rate distribution on the drum surface; three scintillating fibers are also visible.

The mockup geometry of Figure 12 was reproduced in FLUKA [17], including the four SciFi detectors around the drum. Then, 10^9^ decays from the abovementioned ^137^Cs gamma source were simulated, tracked, and scaled to the real source activity, finally producing (i) a 3D map of the expected dose rate distribution and (ii) the deposited energy spectra on the four SciFi fibers. Figure 13a shows the expected dose rate distribution on an XY cross-section at mid-height of the drum (40 cm < Z < 41 cm, Figure 13b). Figure 13c shows the expected dose rate distribution on the drum’s surface in 3D, with three scintillating fibers also visible. Figure 14a depicts the expected dose rate distribution on a YZ cross-section with −3 cm < X < −1 cm, as indicated in Figure 14b. Figure 15a shows the expected dose rate distribution on an XZ cross-section with 1 cm < Y < 3 cm, as indicated in Figure 15b.

**Figure 16:**
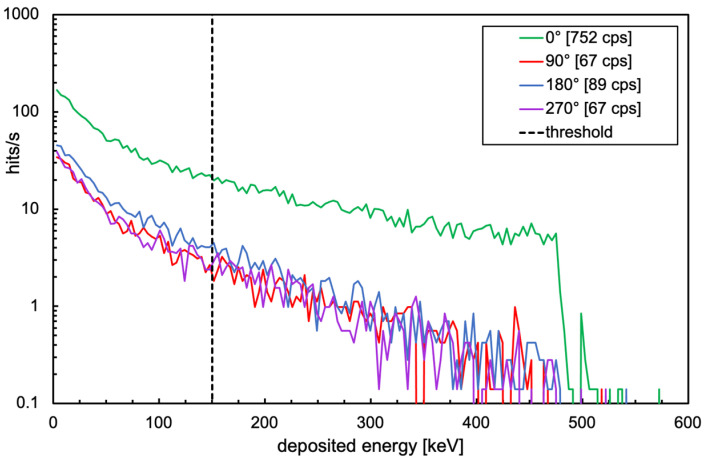
Simulation of the deposited energy spectra for the four SciFi sensors.

Table 1 lists the dose rates measured with a handheld instrument (Thermo FH 40 G-L10) at three different heights in the 0° and 180° positions. The simulated dose rates are also shown for comparison. In light of several unknown experimental conditions (i.e., rough positioning of the handheld instrument for the measurement, approximate positioning of the source inside the mockup, non-perfect geometric features of the concrete mockup, and misalignment between sensors and inner holes), the agreement between simulated values and measured ones looks reasonable. Figure 16 shows the simulated deposited energy spectra for the four SciFi sensors. The expected count rate for each detector, indicated in the legend, was obtained by integrating each spectrum above the selected threshold.

## 3. Results

### 3.1. System Installation

**Figure 17:**
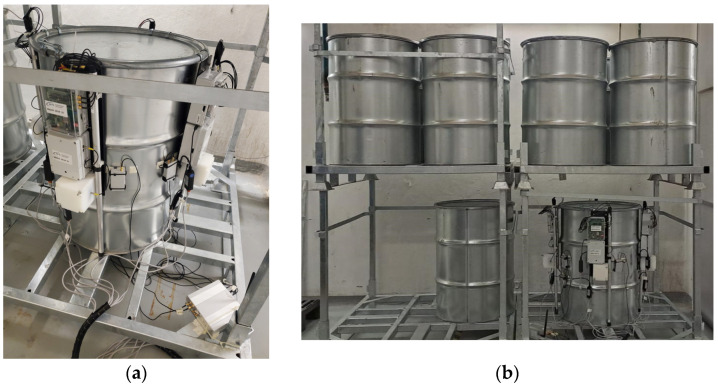
(**a**) The four SciFi sensors hooked to the drum, initially connected via wires for preliminary setup and tuning. (**b**) Several drums, filled with concrete, beside and over the mockup.

**Figure 18:**
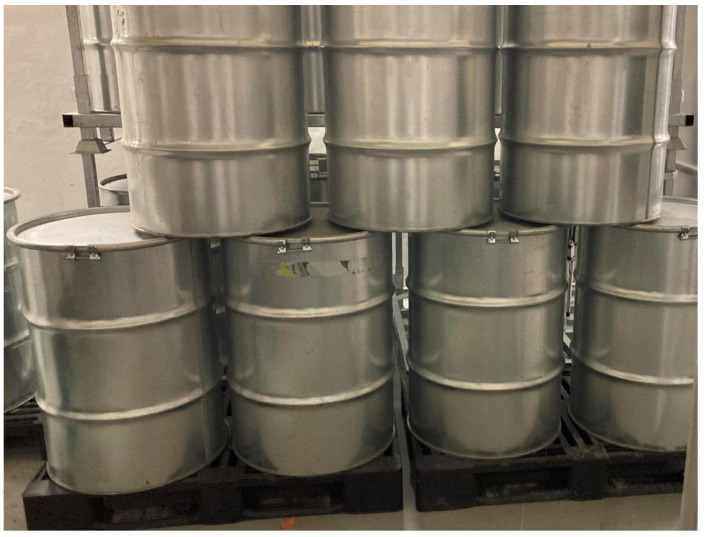
The final demo configuration with other drums filled with concrete placed in front of the mockup.

As already mentioned, due to logistic reasons mainly related to the high source activity and the consequent radiation protection issues, along with the limited access to the storage site and the mockup drum, we decided to run a single demonstration with the radioactive source inserted in the quasi-central hole. The four SciFi sensors were hung on the drum (along with other sensors not discussed in this paper) and initially connected via wires for their preliminary setup and tuning, as shown in Figure 17a. Several drums filled with concrete were placed beside and over the mockup (Figure 17b), and then, the wires were disconnected and other drums with concrete were placed in front (Figure 18) in order to verify the signal transmission capability through such a heavy shielding. An additional SciFi sensor was installed about six meters away in the adjacent room to measure the ambient background. The monitoring units were connected to the private WiFi sub-network, as previously described, and their automatic wake-up was scheduled to occur every six hours, with a measurement time of 60 s. The acquired data were stored locally and sent to the PC/Server and a cloud database for redundant storage.

### 3.2. System Tuning

The sensors’ parameters were preliminarily arranged by roughly comparing the count rate produced by a low-intensity radioactive source with the ambient background. Data were collected over several days while several checks were performed remotely on the sensors’ behavior. The tuning was then refined, setting the voltage bias at the recommended value of 27 V (corresponding to a SiPM overvoltage of about 2.5 V) and choosing a suitable threshold for all the SiPMs. The real data acquisition started on 17 November 2023.

The voltage bias was automatically measured at each sensor wake-up, and its overall average value is reported in Table 2 for the five sensors, along with the uncertainty, given as the standard deviation of all measurements.

In the plots in Figure 3, one can see a peak around channel 350, which is due to the saturation of the amplifier, and this does not represent a problem. Indeed, for the detection of a gamma ray on a SciFi, one simply needs the coincidence of the two signals above the threshold, irrespective of their effective amplitude. Due to the 80 cm fiber length and the λ = 330 cm attenuation length [10], the maximum attenuation *k* experienced by the scintillation light when emitted close to one end is as follows:

(1) $$k=e^{-\frac{80}{330}}\approx 0.78$$

This is the maximum ratio to be expected between the signals from the two up/down SiPMs. Indeed, due to the amplifier saturation, one would expect such a ratio to be mostly close to 1. Typically, when using scintillator bars, one can determine the impact position of the radiation event by event by using Equation (2).

(2) $$ln\frac{B}{A}=ln\frac{Qe^{\left(\frac{L}{2}+z\right)/\lambda }}{Qe^{\left(\frac{L}{2}-z\right)/\lambda }}=ln\left(e^{\frac{2z}{\lambda }}\right)=\frac{2z}{\lambda }\;\;\;\;\;⟹\;\;\;\;\;z=\frac{\lambda }{2}ln\frac{B}{A}$$

where *B* and *A* are the signal amplitudes measured at the two bar ends, *Q* is the initial scintillation light intensity propagating in the two directions, *L* is the bar length, *λ* is the bar attenuation length, and *z* is the hit coordinate with respect to the middle point of the bar [18]. The scintillator bars are usually wrapped with reflective foil in order to maximize the amount of trapped light. Unfortunately, in a scintillating fiber with a round cross-section, the amount of trapped light is ≲6% in each direction [19], and this, combined with the SiPM photon detection efficiency, generally produces signals with few detected photons in response to gamma-ray interactions [7]. Therefore, due to the low photon statistics, the position resolution of a SciFi with such a technique is very poor. Moreover, the amplifier saturation further blurs the information. Nonetheless, from Equations (1) and (2) one would expect the following:

(3) $$\left|ln\left(B/A\right)\right|\leq ln\left(k\right)\approx 0.25$$

**Figure 19:**
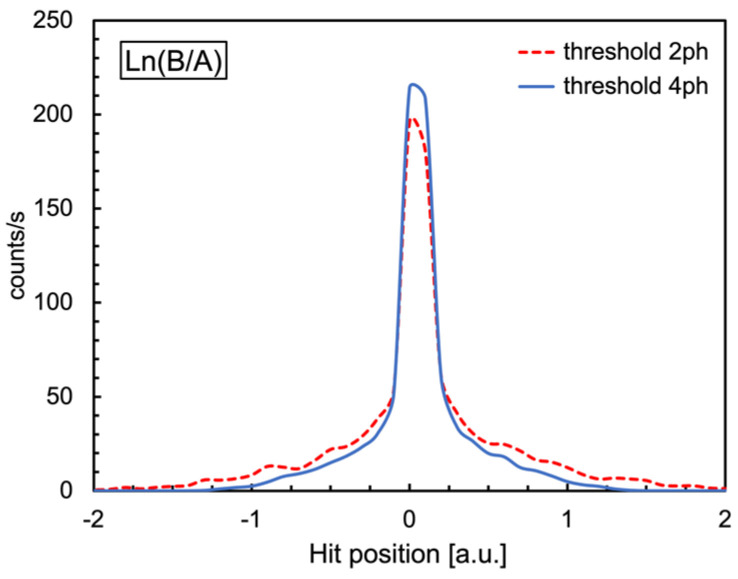
Distribution of the event-by-event natural logarithm of the B/A ratio of Equation (2), with SiPM thresholds at two and four photons, respectively.

This is shown in Figure 19 for events detected by the SciFi at 0° with the SiPM thresholds set at two and four photons. One can easily see that the tails of the distribution, mainly due to the statistical fluctuations when the number of photons is small, are shortened by enforcing a proper threshold. 

### 3.3. Measurement Results

**Figure 20:**
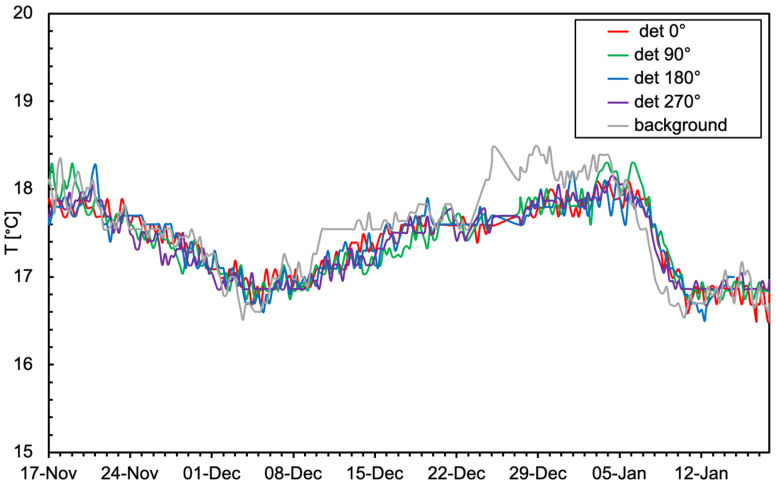
Temperature values measured in the storage room by the five sensors.

At each sensor wake-up, the temperature inside the electronics box was measured. Since the wake-up was occurring every six hours, one can safely assume that the measured values correspond to the ambient temperature. The collection of the measured values during a two-month period is reported in Figure 20 for the five sensors. One can observe small fluctuations, likely to be ascribed to slight daily local changes or limited sensor precision, and a slower overall oscillation due to environmental temperature changes. A few sharp fluctuations observed in some cases could be attributed to electrical noise on the temperature sensors or perhaps to perturbations produced by local operations performed by people in the vicinity of the drums. 

**Figure 21:**
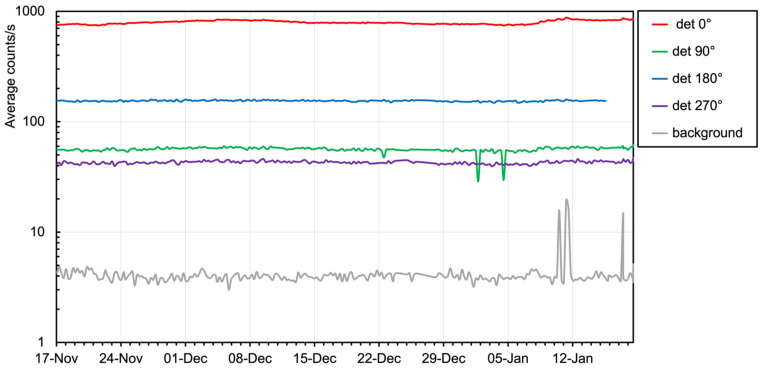
Raw count rate data of the five installed detectors.

**Figure 22:**
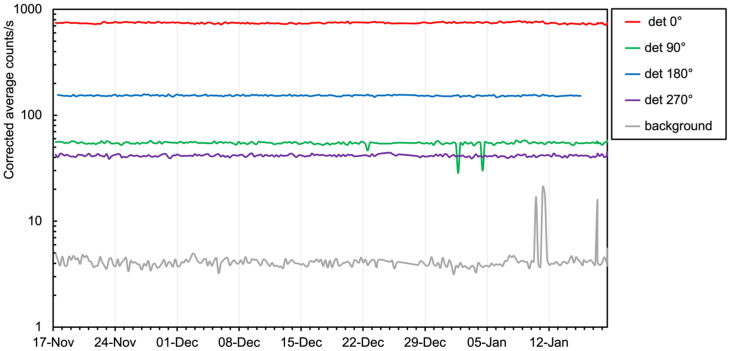
Count rate data after correction for temperature variations (see text for details).

The count rate data of the five installed detectors, averaged over the sixty-second measuring time interval, are reported in Figure 21 as a function of time. As expected, the behavior is relatively flat, but not completely flat because of some slight up-down drifts due to temperature changes. It is well known that the SiPM breakdown voltage, and thus, its gain, has a small dependence on the temperature over a wide interval via a given linear coefficient. However, in the observed temperature range, such a drift should have been negligible. The drift was to be ascribed to the behavior of the analog electronics (mainly the amplifiers) because they were operating at the ambient temperature just after wake-up without reaching thermal equilibrium. Nonetheless, a simplified approach was followed to compensate for the drift: for each SciFi, we used the count rate measured on the first measurement day as a reference and linearly fitted its variation with decreasing temperature in the following fourteen days. The obtained coefficients were used to correct the count rate into an effective one for every measured data point, and the resulting plot is shown in Figure 22. Unfortunately, due to a defective contact on its battery pack, the sensor at 180° stopped working just a few days before the end of the test. We also observed that the background rate (the grey line in the plots) shows three sharp peaks, which can be explained as a pick-up of electrical noise. Indeed, the sensor was hung on a wall beside an electricity distribution panel where some big devices nearby were connected.

The average count rate values were roughly rescaled to corresponding dose rates for the five SciFi detectors following similar considerations as in ref. [7]. We remark that such a rescaling is rough because (i) the sensors are not point-like and (ii) even though the source is point-like, the gamma rays are also scattered by concrete and steel; therefore, the emission geometry is diffuse. As a consequence, the estimated dose rates have to be considered as an average indication as they are distributed over the fiber length. Count and dose rates are listed in Table 3. 

**Figure 23:**
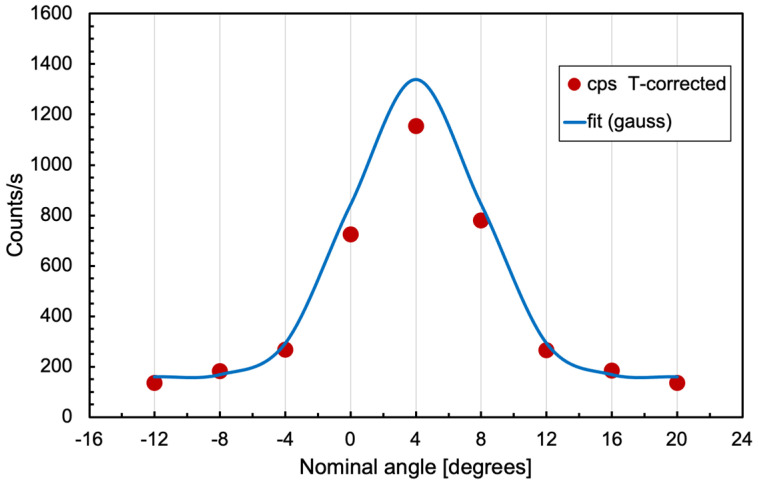
Count rate data acquired in several positions around the nominal 0°.

Before concluding the demonstration, we acquired data in several angular positions by displacing the 0° sensor around the drum, thus discovering that its nominal position (and the other three) was shifted by about 4° from the initially assumed one in front of the rectangular hole. This is clearly visible in Figure 23. The positions used for the simulation, and indicated in Figure 12, take into account such a displacement.

## 4. Conclusions

The predisposal treatment of radioactive waste streams other than nuclear fuel and high-level radioactive waste usually involves a thorough radiological characterization of the packed drums. The time lapses before and after characterization could be somewhat long; therefore, monitoring the drums during these phases could be useful for early discovery of possible anomalies. Due to its wireless and battery operation mode, as well as its hookwise mechanical fixing, the SciFi sensor has shown to be quickly and easily installed, positioned, displaced, and removed. Its programming flexibility makes it possible to employ it for almost continuous measurements in the short term, useful for radiation protection purposes, as well as for longer-term daily measurements to check the overall integrity of drums in view of their final disposal. Tampering with, or unauthorized operations on, the drums could be promptly detected as a sharp behavioral change of the count rate(s), therefore providing an additional security benefit on top of the safety one. The acquired data are stored locally on the SD memory card of each electronics box and the PC/server. They are also automatically transmitted to a cloud database for permanent archival storage, thus ensuring data integrity because of the multiple redundancy. We showed that a number of sensors arranged around a drum (in the present case, four units) provide indications of the emission anisotropy, with any sensitive change in the measured count rate hinting at some anomaly and possibly triggering a suitable inspection by operators. 

The forthcoming activity with the sensors will be focused on the characterization of their anomaly detection capability by simulating several configurations like tiny cracks on the steel skin and/or on the concrete bulk and by planning specifically designed laboratory tests. An already planned improvement of the electronics will be the implementation of LoRa [20], a better-performing lower-power and longer-range wireless connection.

Even though the initial proposal in the PREDIS project was to monitor the gamma radioactivity from cemented radwaste drums, we are convinced that the SciFi devices described in this paper could be usefully employed in a wider range of applications. Indeed, we are currently planning to employ them as low-cost solutions for the safety/security of rooms and people in public places and to equip tents for in-field nuclear safeguards operations.

## Figures and Tables

**Table 1:** Simulated and measured dose rates at three different heights in the 0° and 180° positions around the drum. Also reported are the dose rates evaluated by means of the corresponding SciFi sensors (averaged along the length). See Section 3.3.

Position	Dose Rate at 0° [µSv/h]			Dose Rate at 180° [µSv/h]		
	Simulated	Measured	SciFi	Simulated	Measured	SciFi
high	30	45	32	2	1.5	6.6
middle	115	80		18	15	
low	30	25		2	3	

**Table 2:** The voltage bias value set and the corresponding measured one for the five sensors.

	Det 0°	Det 90°	Det 180°	Det 270°	Det Background
set Vbias	27	27	27	27	27
measured Vbias	27.00 ± 0.027	27.15 ± 0.026	26.95 ± 0.028	27.01 ± 0.036	26.86 ± 0.021

**Table 3:** Average count rates and (roughly) corresponding dose rates for the five SciFi detectors.

SciFi	Average Count Rate Measured [cps]	Average Count Rate Simulated [cps]	Average Dose Rate from Measurement [µSv/h]	Average Dose Rate from Simulation [µSv/h]
0°	745.0	752	32	32
90°	55.0	67	2.4	2.9
180°	153.8	89	6.6	3.8
270°	41.6	67	1.8	2.9
Background	4.1	-	0.2	-

## Data Availability

The data presented in this study are available upon request from the corresponding author.

## References

[1] PREDIS Project, Euratom H2020, GA No. 945098. https://predis-h2020.eu/.

[2] IAEA (2014). Monitoring and Surveillance of Radioactive Waste Disposal Facilities.

[3] Finocchiaro P. (2011). DMNR: A new concept for real-time online monitoring of short and medium term radioactive waste. Radioactive Waste: Sources, Types and Management.

[4] Cosentino L., Calì C., De Luca G., Guardo G., Litrico P., Pappalardo A., Piscopo M., Scirè C., Scirè S., Vecchio G. (2012). Real-Time Online Monitoring of Radwaste Storage: A Proof-of-Principle Test Prototype. IEEE Trans. Nucl. Sci..

[5] Finocchiaro P., Ripani M. Radioactive Waste Monitoring: Opportunities from New Technologies. Proceedings of the IAEA International Conference on Physical Protection of Nuclear Material and Nuclear Facilities, IAEA-CN-254/117.

[6] Cosentino L., Giuffrida M., Lo Meo S., Longhitano F., Pappalardo A., Passaro G., Finocchiaro P. (2021). Gamma—Ray Counters to Monitor Radioactive Waste Packages in the MICADO Project. Instruments.

[7] Cosentino L., Ducasse Q., Giuffrida M., Lo Meo S., Longhitano F., Marchetta C., Massara A., Pappalardo P., Passaro G., Russo S. (2021). SiLiF Neutron Counters to Monitor Nuclear Materials in the MICADO Project. Sensors.

[8] MICADO Project. https://www.micado-project.eu/.

[9] Han Y., Xu S., Huang Y. (2022). Real-Time Monitoring Method for Radioactive Substances Using Monolithic Active Pixel Sensors (MAPS). Sensors.

[10] Luxium Solutions (Previously Saint Gobain Crystals) Scintillating Fibers. https://www.mi-net.co.uk/product/scintillating-fiber/.

[11] ON Semiconductor. https://www.onsemi.com/products/sensors/photodetectors-sipm-spad/silicon-photomultipliers-sipm.

[12] Dolgoshein B., Balagura V., Buzhan P., Danilov M., Filatov L., Garutti E., Groll M., Ilyin A., Kantserova V., Kaplin V. (2006). Status report on silicon photomultiplier development and its applications. Nucl. Instrum. Meth. A.

[13] Zappa F., Tisa S., Tosi A., Cova S. (2007). Principles and features of single-photon avalanche diode arrays. Sens. Actuators A.

[14] Finocchiaro P., Pappalardo A., Cosentino L., Belluso M., Billotta S., Bonanno G., Carbone B., Condorelli G., Di Mauro S., Fallica G. (2008). Characterization of a Novel 100-Channel Silicon Photomultiplier—Part I: Noise. IEEE Trans. Electron. Devices.

[15] Finocchiaro P., Pappalardo A., Cosentino L., Belluso M., Billotta S., Bonanno G., Carbone B., Condorelli G., Di Mauro S., Fallica G. (2008). Characterization of a Novel 100-Channel Silicon Photomultiplier— Part II: Charge and Time. IEEE Trans. Electron. Devices.

[16] Espressif. https://www.espressif.com/sites/default/files/documentation/esp32_datasheet_en.pdf.

[17] Fasso A., Ferrari A., Ranft J., Sala P.R. (2005). FLUKA: A Multi-Particle Transport Code.

[18] Cosentino L., Finocchiaro P., Pappalardo A., Garibaldi F. (2012). Characterization of a scintillating mini-detector for time-of- flight positron emission tomography with depth-of-interaction. Rev. Sci. Instrum..

[19] Papandreou Z. (2007). Scintillating Fiber Trapping Efficiency, GlueX-doc-918-v2.

[20] Paul B. (2020). An Overview of LoRaWAN. WSEAS Trans. Commun..

